# Cold tolerance strategy and cryoprotectants of *Megabruchidius dorsalis* in different temperature and time stresses

**DOI:** 10.3389/fphys.2022.1118955

**Published:** 2023-01-11

**Authors:** Si-Yu Chen, Ru-Na Zhao, You Li, He-Ping Li, Ming-Hui Xie, Jian-Feng Liu, Mao-Fa Yang, Cheng-Xu Wu

**Affiliations:** ^1^ College of Forestry, Guizhou University, Guiyang, Guizhou, China; ^2^ Guizhou Provincial Key Laboratory for Agricultural Pest Management of the Mountainous Region, Scientific Observing and Experiment Station of Crop Pest Guiyang, Ministry of Agriculture, Institute of Entomology, Guizhou University, Guiyang, China; ^3^ Fujian Province Key Laboratory of Plant Virology, Fujian Agriculture and Forestry University, Vector-Borne Virus Research Center, Fuzhou, China; ^4^ College of Tobacco Science, Guizhou University, Guiyang, China

**Keywords:** *Megabruchidius dorsalis*, temperature acclimation, supercooling point, freezing point, cold-resistant substances

## Abstract

The honey locusts (genus *Gleditsia*) are a genus of high-value trees in Asia. Seed beetle, *Megabruchidius dorsalis* (Fåhraeus) (Col.: Chrysomelidae: Bruchinae), is a *Gleditsia* oligophagous pest that causes severe yield reduction. To understand the cold tolerance of *M. dorsalis* adults, this study investigated its cold tolerance strategy and the influence of low temperatures on its physiology and biochemistry. The low-temperature treatments were divided into three groups: long-term temperature acclimation (Group 1; 15°C, or 20°C, or 25°C, or 28°C [control check, CK] for 10 days), short-term low-temperature exposure (Group 2; 0°C or 4°C for 2 h), and long-term low-temperature induction (Group 3; 0°C or 4°C for 1, 3, or 5 d). The supercooling point (SCP; temperature at which spontaneous nucleation and ice lattice growth begin), freezing point (FP; temperature at which insect fluids freeze), low lethal temperature (LLT; temperature at which all individuals are killed), water, lipid, glycerol, and total sugars contents were measured under different temperature stresses. The results showed that *M. dorsalis* adults were a freeze-avoidant species. The SCP and LLT at 28°C were −10.62°C and −19.48°C, respectively. The SCP and FP of long-term temperature acclimation (15°C, or 20°C, or 25°C) were significantly lower than that of the control group (28°C). The water content of the long-term low temperature induction (0°C) group was significantly lower than that of the control group. The lipid and glycerol content in the acclimated group at 20°C and 25°C were significantly higher than in the control group. *M. dorsalis* adults may maintain their biofluids in a supercooled state *via* cryoprotectant accumulation and cryoprotective dehydration to prevent ice nucleation. This study provides a theoretical basis for future research on overwintering and potential distribution and related prediction of *M. dorsalis* adults.

## 1 Introduction

*Gleditsia* is well known for its significant medicinal uses and biological activity, which is a high-value tree species (Ragab et al., 2021). Its seed can be made into edible “snow lotus seeds” (*Gleditsia* kernels) in China. *Megabruchidius dorsalis* (Fåhraeus) (Col.: Chrysomelidae: Bruchinae) is an oligophagous seed pest, mainly infesting *Gleditsia* Lam. plants (Fabaceae), overwintering as 4th instar larvae and adults in the pod (Ruta et al., 2017; Pintilioaie et al., 2018), which is distributed in Asia and Europe (György and Tuda, 2020). The female adults of *M. dorsalis* deposit eggs on the pods and the larvae hatch and burrow into the seeds until adult emergence (Shimada et al., 2001). The majority of *Gleditsia* seeds will be bored, causing serious economic losses (Lezhenina and Vasilieva, 2020).

Insects are ectothermic animals. The environmental temperature directly affects their adaptability and physiological processes, meaning that temperature affects their survival and distribution (Leather et al., 1993; Régnière et al., 2012; Jaworski and Hilszczański, 2014). Cold hardiness refers to the ability of insects to cope with long- or short-term exposure to low temperatures (Sinclair et al., 2015). The supercooling point (SCP; the temperature at which spontaneous nucleation and ice lattice growth begin) and freezing point (FP; the temperature at which insect fluids freeze) in insects are key markers of their cold tolerance (Lee, 1989; Vétek et al., 2020). The low lethal temperature (LLT; the temperature at which all individuals are killed) and the LT_50_ (median lethal temperature, expected to kill 50% of a population) can be used to estimate the absolute limit of insect survival at low temperatures (Sinclair et al., 2015). Based on SCPs, the cold tolerance strategies of insects can be divided into three main categories: chill susceptibility (mortality occurs without internal ice formation, LT_50_>SCP), freeze avoidance (survival without internal ice formation and death with internal ice formation, LT_50_ = SCP) and freeze tolerance (tolerate the formation of ice in their body tissues and fluids, LT_50_ < SCP) (Slabber and Chown, 2004; Bemani et al., 2012; Sinclair et al., 2015). Insects can adjust their SCP to cope with cold stress accompanying latitude increases. For example, Xie et al. (2015) discovered that tropical populations of *Ostrinia furnacalis* had higher SCPs than temperate populations, and warm temperate populations were mainly intermediate between temperate and tropical populations.

Insects adapt to cold temperatures through physiological and biochemical adjustments in the body. When exposed to low temperatures or overwintering, insects may limit their activity and metabolism by regulating lipids and carbohydrate contents (Arrese and Soulages, 2010; Sinclair and Marshall, 2018; Hasanvand et al., 2020). Low molecular weight carbohydrates and polyols are important cryoprotectants (Fuller, 2004). The SCP of *Anoplophora glabripennis* was negatively correlated with the contents of low molecular weight sugars (glucose and mannitol) and glycerol, implying that *A. glabripennis* decreased their SCP and enhanced cold resistance by increasing the concentrations of these compounds in winter (Feng et al., 2016). Cryoprotective dehydration is one of the strategies that insects adapt to cold conditions (Clark et al., 2009; Guillén et al., 2022), namely by reducing water content and increasing biofluid concentrations to enhance their supercooling ability, or reducing damage from freezing of biofluids (Zachariassen, 1985; Zhao et al., 2019; Pei et al., 2020).

Rapid cold hardening (after minutes to about 3 h of exposure to low temperatures) can affect a range of cold hardiness parameters, and cold acclimation (days to weeks) also has a significant effect on cold tolerance (Teets and Denlinger, 2013; Jakobs et al., 2015). According to the beneficial acclimation hypothesis, the association of insect adaptive acclimation with SCP can support the cold limit, which is related to acclimation temperature and exposure time (Hemmati et al., 2014; Noor-Ul-ane and Jung, 2021). The SCP of *Phthorimaea operculella* (Aryal and Jung, 2018) and *Helicopter assulta* (Cha and Lee, 2016) decreased significantly after cold acclimation. Insects exposed to cold acclimation before overwintering in natural circumstances improve their capacity to withstand freezing temperatures (Wu et al., 2016).

**FIGURE 1:**
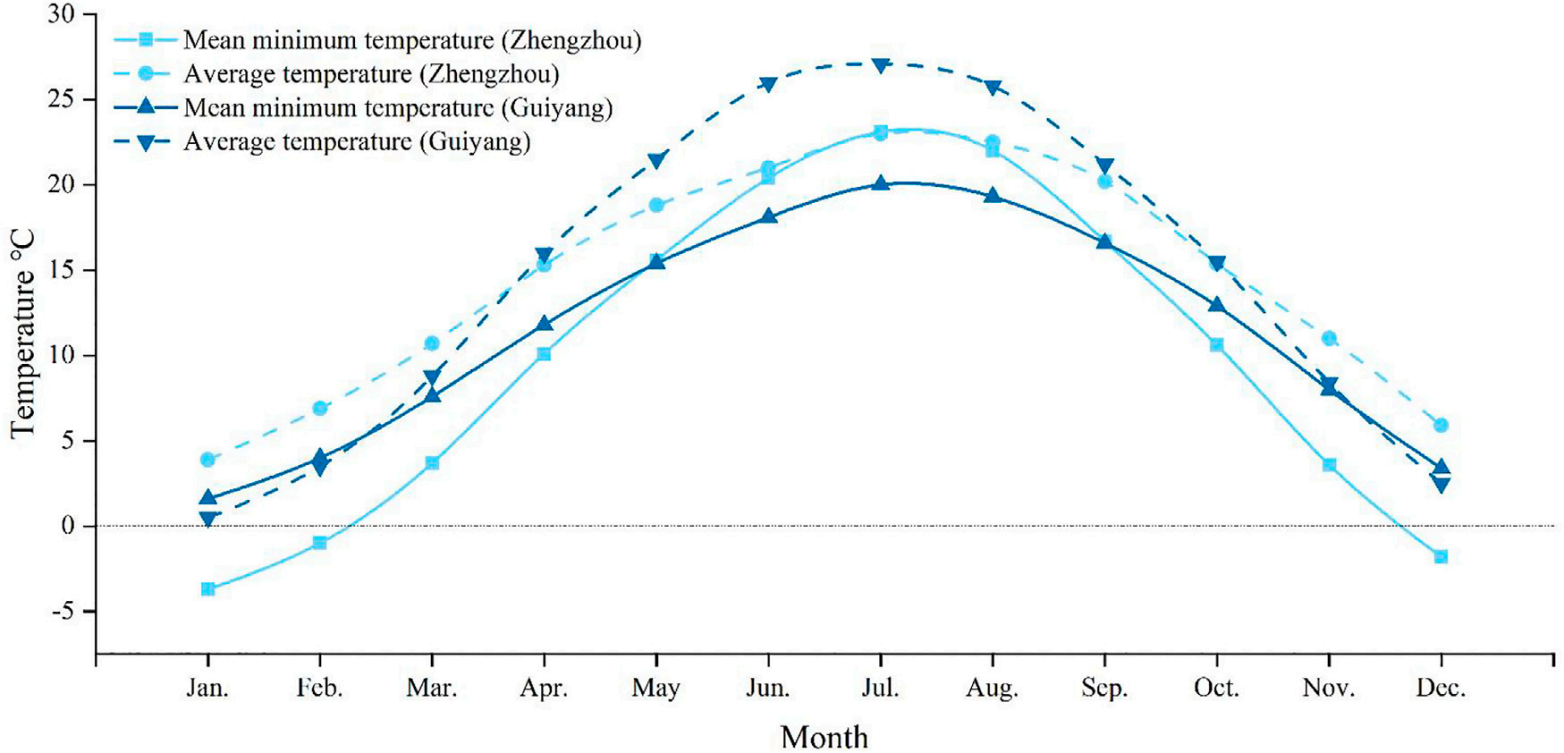
The monthly average temperature and monthly mean minimum temperature in Guizhou (Guiyang) and Henan (Zhengzhou) provinces from 1981 to 2010. The data comes from China Meteorological Data Service Centre (http://data.cma.cn/).

Understanding insect cold tolerance and variations in their content of cold tolerance-related substances are critical for determining the occurrence trends, distribution range, and overwintering limits (Prasanna et al., 2018). In China, the damage caused by *M. dorsalis* in Guizhou Province (Guiyang: 106°37′48″E 26°38′N) is more severe than that in Henan Province (Zhengzhou: 113°37′ E, 34°44′ N). Based on this phenomenon and temperature differences between these two provinces (Figure 1), the supercooling point, freezing point, water content, lipid content, glycerol content, and total sugar content of *M. dorsalis* adults were measured under conditions of long-term temperature acclimation (15°C, 20°C, 25°C, 28°C), short-term low-temperature exposure (0°C, 4°C; 2 h), and long-term low-temperature induction (0°C, 4°C; 1, 3, or 5 d), and the low lethal temperature of *M. dorsalis* was also determined at 28°C. These results preliminarily determined the cold tolerance of this insect and provided a theoretical basis for studying the potential distribution and correlation predictions for *M. dorsalis*.

## 2 Materials and methods

### 2.1 Insects

Adults of *M. dorsalis* were collected in October 2020 from *Gleditsia sinensis* Lam. in the vicinity of Zhijin County, Guizhou Province, China (106°03″–106°04″E, 26°32″–26°33″ N) and reared on seeds of *G. sinensis* in plastic boxes (225 × 155 × 80 mm) in a rearing incubator (RXZ–380A–LED, Ningbo Jiangnan Instrument Factory, Ningbo, China) at Forest Conservation Laboratory in the College of Forestry, Guizhou University. The incubator was maintained at 28°C ± 1°C and 65 ± 5% relative humidity with a 14:10-h dark:light photoperiod. All tested samples were continuously bred for more than three generations in plastic finishing boxes that were covered with gauze. After adults emerged, the beetles were separated by sex for further testing according to the morphological characteristics described by Li et al. (2012) and fed with fresh cucumber slices *ad libitum* daily.

### 2.2 Determination of SCP and FP and cold tolerance

We assessed the effects of low temperatures with different temperatures and time stresses on the cold tolerance of *M. dorsalis*. Healthy adults from the same batch (within 72 h) with uniform size were selected for experiments, and the treatments were divided into three groups: long-term temperature acclimation (Group 1), short-term low-temperature exposure (Group 2), and long-term low-temperature induction (Group 3). In Group 1, the adults were reared at 15°C, or 20°C, or 25°C, or 28°C (control check, CK) for 10 days. In Group 2, the adults were exposed to temperatures of 0°C or 4°C for 2 h. In Group 3, the adults were induced at 0°C or 4°C for 1, 3, or 5 d. Control adults were kept at 28°C, and the feeding conditions were unchanged in this group. All tested adults were alive before the experiments. In all treatments, male and female adults were separated and placed on filter paper in Petri dishes (9 cm diameter), and then they were placed in the rearing incubator (Ningbo Jiangnan Instrument Factory, Ningbo, China) or a refrigerator (BCD-576WDPU; Haier, Qingdao, China). The range of temperature fluctuations was ±1°C.

The SCP and FP were measured using the thermocouple method (SUN-V Cold Point For Insect, Peng Cheng Electronic Technology Center of Beijing, China) as described by Sinclair et al. (2015). The abdomens of adults were fixed on a temperature-sensitive probe with transparent tape. The fixed insect bodies and the probe were wrapped with absorbent cotton to prevent rapid cooling of the body and placed together in a low-temperature test box (DW-40L525; Aucama, Qingdao, China) with a constant temperature of -30°C; the temperature was lowered continuously at the rate of 1°C/min. Thirty adults (♀:♂ = 1:1) were measured at each temperature, and data were automatically recorded using the software.

### 2.3 Low lethal temperature

Healthy adults from the same batch (within 72 h) with uniform size were selected for experiments. Twenty adults (♀:♂ = 1 : 1) were placed on filter paper in Petri dishes (90 mm diameter) and held at lower ranges with seven temperature gradients (−8°C, −10°C, −12°C, −14°C, −16°C, −18°C, or −20°C [resulting in mortality from 0% to 100%]) for 1 hour (DPMS-358, Ningbo Jiangnan Instrument Factory, Ningbo, China). After treatment, *M. dorsalis* adults were placed back in an incubator at normal temperature (25°C) and mortality was evaluated by observing behavioral responses when touched with a small brush (no response considered death). Each treatment was repeated five times. The LLT and LT_50_ were estimated using logistic regression in SAS software (v9.4; SAS Institute, Cary, NC, United States) as described by Sinclair et al. (2015). Logistic regression equation:

$$M\left(T\right)=e^{\left(a+b\times T\right)}/\left(1+e^{\left(a+b\times T\right)}\right)$$ (1)

*T* is the temperature of the insect’s environment. M(*T*) is the mortality of insects at temperature *T*. The a and b are the coefficients of the logistic regression Equation 1. First, the experimental data were inserted into a logistic regression Equation 1 to estimate the parameters a and b, then the known mortality rate was 50% and 100%, and the corresponding temperature was calculated.

### 2.4 Water and lipid contents

After the same pretreatment as in 2.2, according to the method of Song (2017), 1.5-mL centrifuge tubes were numbered and weighed (W) using an electronic balance (d = 0.0001 g, PR124 ZH/E; Ohouse Instruments [Changzhou] Co., Ltd., Changzhou, China). Four adults (♀:♂ = 1:1) were placed into tubes and their wet weight (fresh weight, FW) was measured. After being placed in an oven (WGL-30B; Taisite, Tianjin, China) at 60°C for 48 h, the dried weight (dry weight, DW) was measured. Five 2-mm grinding beads (bead weight, BW) and 0.2 mL of a 2:1 chloroform–methanol were added into the tube. The insect bodies were crushed using a fully automatic sample freeze-grinding instrument (JXFSTPRP-CL; Jingxin, Shanghai, China), and 0.8 mL of the chloroform–methanol mixture were added. Samples were mixed and centrifuged for 10 min (10000 rpm; Centrifuge 5418 R; Eppendorf, Hamburg, Germany). After removing the supernatant, 1 mL of the chloroform–methonal mixture was added. Centrifuge tubes with residue were placed in an oven (60°C) for 24 h to determine the lean dry weight (LDW). Water content measurements were repeated six times, and lipid content measurements were repeated five times.

$$Water\;content\;\left(\%\right)=\left[\frac{\left[\left(FW-W\right)-\left(DW-W\right)\right]}{FW-W}\right]\times 100$$

$$Lipid\;content\;\left(\%\right)=\left[\frac{\left[\left(DW-W\right)-\left(LDW-W-BW\right)\right]}{DW-W}\right]\times 100$$

### 2.5 Glycerol content

After the same pretreatment as in 2.2, the methods of Song (2017) and Huang (2014) were adopted to make the oxidant, reducing agent (color developer), glycerol standard solution, and standard curve. A 1.5-mL centrifuge tube was weighed (W1) and four treated adults were added and weighed (♀:♂ = 1:1; W2). Five 2-mm grinding beads were added with 200 µL pure water, and the mixture was ground in the tube using a frozen grinding instrument. Then, 1300 µL of pure water were added and mixed thoroughly. Samples were centrifuged (10000 rpm, 10 min) and the supernatant was removed. The oxidation, color development, and optical density (OD) value of the solution were measured, and the corresponding glycerol content was obtained from the standard curve. Measurements were repeated six times per treatment.

$$Glycerol\;content\;\left(\frac{g}{mg}\right)=\left[\frac{\left[determination\;vaules\;\left(\frac{g}{mL}\right)\times sample\;dilution\;\left(mL\right)\right]}{adults\;weight\;\left(W2-W1\right)\;\left(mg\right)}\right]$$

### 2.6 Total sugar content

After the pretreatment described in 2.1, the total sugar content was measured using a total sugar content assay kit (ZT-2-Y; Suzhou Comin biotechnology Co., Ltd., Suzhou, China) by sampling four adults (♀:♂ = 1 : 1). The adults were weighed with an electronic balance and placed into centrifuge tubes. Then, 0.5 mL of Reagent I and 0.75 mL of distilled water were added, and the mixture was heated in a 95°C water bath for 30 min, before adding 0.5 mL of Reagent II and mixing. The volume was adjusted to 5 mL with distilled water and the tubes were centrifuged (12000 rpm) at 25°C for 10 min. The supernatant was removed, and 40 μL of supernatant was added to 60 μL of reagent III and 40 μL distilled water for determination tubes, and 80 μL of distilled water was added to 60 μL of reagent III as the control tube. Tubes were mixed and heated in a 95°C water bath for 10 min. Then, the final volume was adjusted to 860 μL with distilled water. The absorbance at 540 (A540) nm was then measured using a spectrophotometer (TU-1901; Beijing Persee General Instrument Co., Ltd., China). Measurements were repeated 3–5 times per treatment. The total sugar content was calculated using the following equation:

$$Total\;sugar\;content\left(\frac{mg}{g}\right)=\left[\frac{\left[22.207\times \left(determination\;A-control\;A+0.0507\right)\right]}{fresh\;weight}\right]$$

### 2.7 Statistical analyses

The data were processed using SPSS Statistics 26.0 (IBM Corp., Armonk, NY, United States), and an independent-samples *t*-test or a One-Way ANOVA was used to analyze the data. The Tukey method was used for multiple comparisons, and the Kruskal–Wallis test was used as a non-parametric test for data non-normal distribution or uneven variance. Origin 2018 software (OriginLab Corp., Northampton, MA, United States) was used for mapping. Data are reported as mean ± SE, level of significance in all tests was set at *p* < 0.05. A correlation analysis of trends among mean SCP, temperature, water content, lipid content, glycerol content, and total sugar content was performed using the Spearman method (each n = 5).

## 3 Results

### 3.1 Supercooling point and freezing point

#### 3.1.1 Sexual differences

**FIGURE 2:**
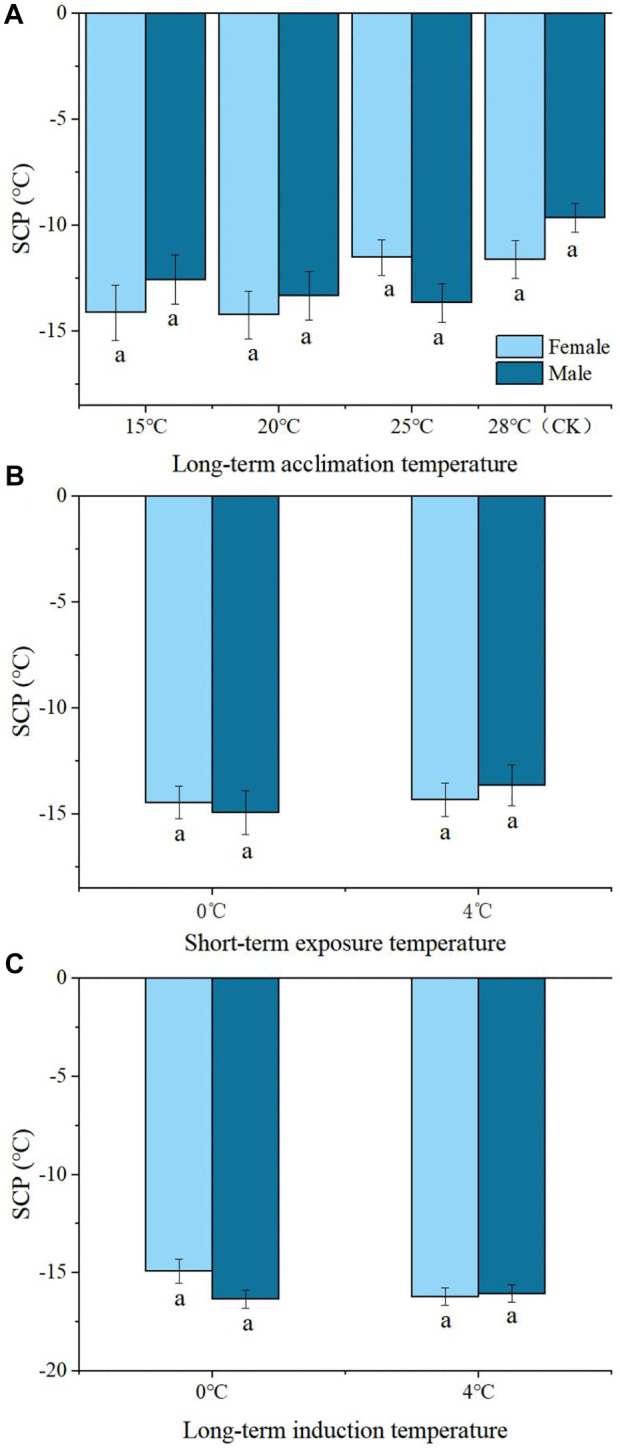
The difference of SCP of *Megabruchidius dorsalis* adults under different treatments **(A)**, long-term acclimation temperature; **(B)**, short-term exposure temperature; **(C)**, long-term induction temperature). At the same temperature, the same lowercase letters indicate no significant difference between males and females, the difference between male and female were tested by independent-samples *t*-test (*p* < 0.05).

There were no significant differences in SCP between male and female adults at 15°C, or 20°C, or 25°C, or 28°C of Group 1 (Figure 2A). Likewise, there was no significant difference in SCP between male and female adults at 0°C or 4°C for 2, or 1, or 3, or 5 d of Group 2 or Group 3. (Figures 2B, C).

#### 3.1.2 Temperature or time difference

The results from the control group (28°C) showed that the SCP was −10.62°C ± 0.63°C and the FP was −8.66°C ± 0.58°C. The SCP of adults acclimated to temperatures from 15°C to 28°C (CK) increased with temperature increases (Table 1). At 20°C, the FP was significantly lower than that at 28°C (*df* = 3, *p* = 0.015) (Table 1). After a short period of exposure to low temperatures, the SCP and FP at 0°C (SCP: *df* = 2, *p* = 0.000; FP: *df* = 2, *p* = 0.000) and 4°C (SCP: *df* = 2, *p* = 0.001, FP: *df* = 2, *p* = 0.003) were significantly lower than those at 28°C (Table 1). The long-term low-temperature induction group SCP and FP at 0°C (SCP: *df* = 2, *p* = 0.000; FP: *df* = 2 *p* = 0.000) and 4°C (SCP: *df* = 2, *p* = 0.001, FP: *df* = 2, *p* = 0.003) were significantly lower than those at 28°C (Table 1).

**TABLE 1:** Supercooling point and freezing point of female and male adults of *Megabruchidius dorsalis* at different treatments.

Treatments	Times	Temperature (°C)	Gender	SCP (°C)			FP (°C)		
				Mean ± SE	Max	Min	Mean ± SE	Max	Min
CK Control check	<72 h	28	♀+♂	−10.62 ± 0.58	−5.81	−15	−8.66 ± 0.66	−3.15	−12.59
Group 1 long-term temperature acclimation	10 d	15	♀+♂	−13.34 ± 0.86	−5.22	−21.47	−11.39 ± 0.78	−3.95	−18.38
		20	♀+♂	−13.38 ± 0.84	−3.39	−22.14	−11.82 ± 0.77*	−2.17	−18.71
		25	♀+♂	−12.53 ± 0.65	−6.78	−20.82	−10.81 ± 0.64	−4.62	−18.52
Group 2 short-term low temperature exposure	2 h	0	♀+♂	−14.70 ± 0.70*	−6.14	−23.15	−12.74 ± 0.64*	−5.05	−17.86
		4	♀+♂	−14.06 ± 0.56*	−8.7	−18.85	−11.96 ± 0.53*	−6.66	−15.53
Group 3 long-term low temperature induction	1 d	0	♀+♂	−15.51 ± 0.66*	−7.06	−22.04	−13.33 ± 0.58*	−5.26	−18.64
		4	♀+♂	−15.25 ± 0.51*	−8.17	−19.61	−12.96 ± 0.69*	−7.22	−17.31
	3 d	0	♀+♂	−16.32 ± 0.65*	−7.06	−22.04	−14.06 ± 0.49*	−7.19	−19.10
		4	♀+♂	−16.03 ± 0.58*	−9.66	−20.33	−14.18 ± 0.51*	−8.63	−18.74
	5 d	0	♀+♂	−15.09 ± 0.68*	−7.37	−20.47	−12.91 ± 0.58*	−6.95	−17.77
		4	♀+♂	−17.14 ± 0.51*	−9.60	−20.64	−14.41 ± 0.60*	−7.30	−20.56

Note: The asterisk (*) indicated a significant difference between different treatments and control check (Kruskal-wallis test, *p* < 0.05). There are some missing data in Group1, e.g. 20°C: 14 ♀; 25°C: 13 ♀, 12 ♂; 28°C: 10 ♀, 10♂.

The results of the long-term low-temperature induction showed that the SCP and FP were significantly lower at 0°C and 4°C than those at 28°C over 1, 3, and 5 d, respectively (0°C: SCP: *df* = 3, *p* = 0.000; FP: *df* = 3, *p* = 0.000; 4°C: SCP: *df* = 3, *p* = 0.000; FP: *df* = 3 *p* = 0.000). However, there were no significant time differences in the SCP and FP of adults induced at 0°C or 4°C for 1, 3, or 5 d, respectively (Table 1).

### 3.2 Low lethal temperature

**FIGURE 3:**
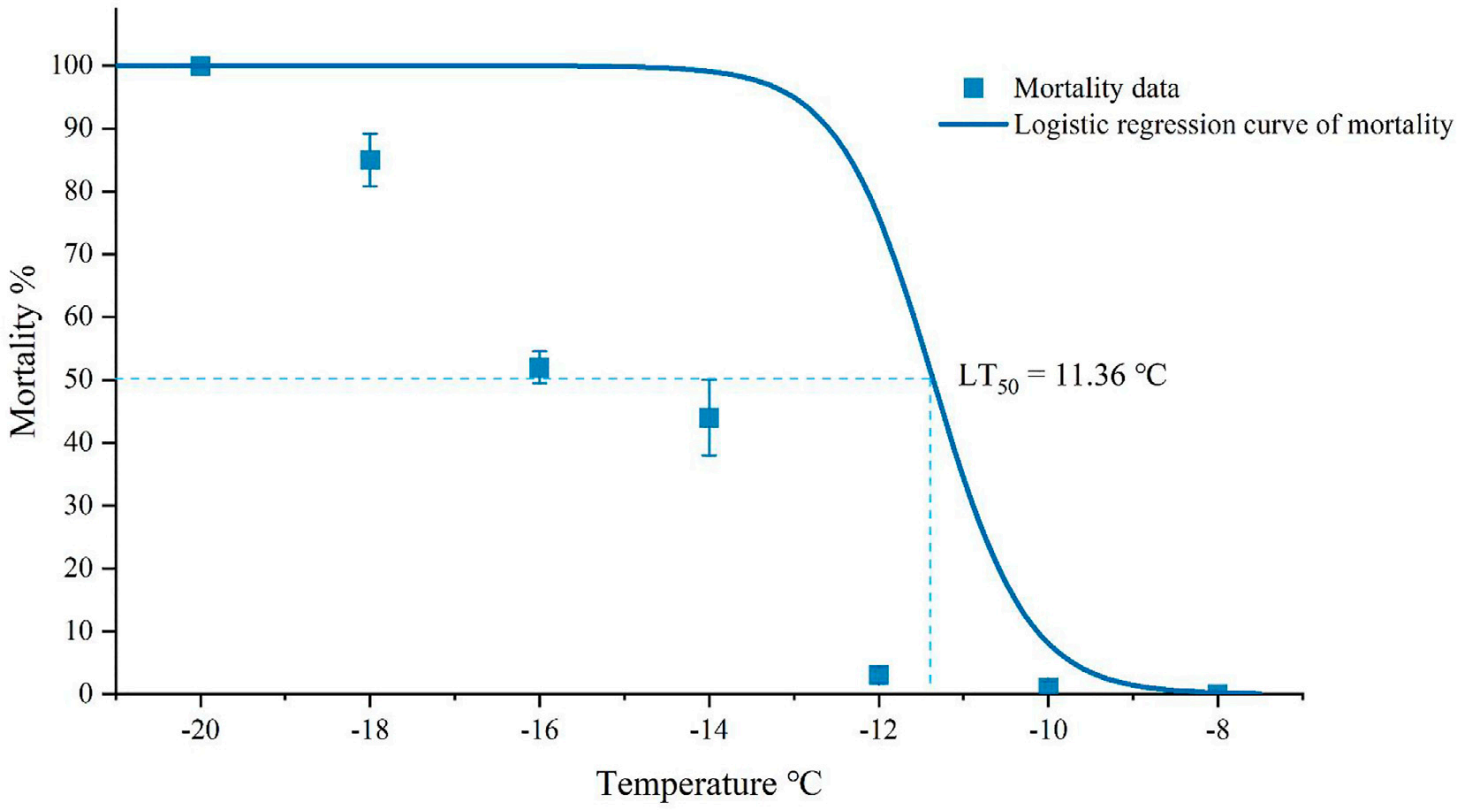
Mortality of *Megabruchidius dorsalis* adults after acute (1 hour) low-temperature exposure. The data were Mean ± SE, and lower lethal temperature and LT_50_ were estimated using logistic regression (*p* < 0.05).

The mortality rates of adults in different temperatures could be well-fitted using logistic regression Equation 1. The constant a of Equation 1 was -20.3215 (*χ* ^*2*^ = 23.9333, *p* < 0.0001), and the linear coefficient b was -1.7889 (*χ* ^*2*^ = 26.2239, *p* < 0.0001) (Figure 3). According to the results of the logistic regression, the LLT was estimated to be about -19.48°C and the LT_50_ was about -11.36°C (Figure 3).

### 3.3 Water and lipid contents

**FIGURE 4:**
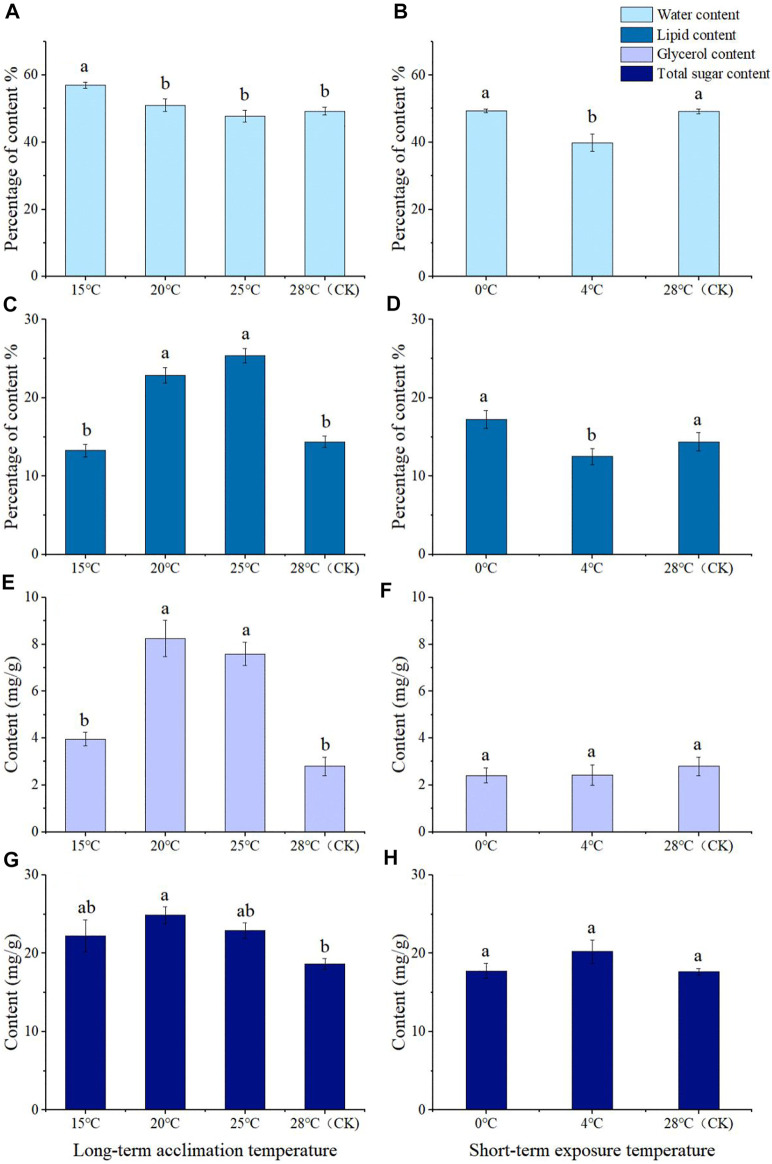
Water content **(A,B)**, lipid content **(C,D)**, glycerol content **(E,F)**, and total sugar content **(G,H)** of *Megabruchidius dorsalis* adults after long-term domestication **(A,C,E,G)** or short-term exposure **(B,D,F,H)**. The data were Mean ± SE, and the different lowercase letters indicated a significant difference among temperature treatments under the same physiological indexes (Kruskal–Wallis test, One-Way ANOVA, Tukey, *p* < 0.05).

The long-term acclimation showed that the water content of *M. dorsalis* adults at 15°C was significantly higher than that of the 28°C (control group), 20°C, and 25°C (*F* = 22.436, *df* = 3, *p* = 0.000), but other groups showed no significant differences in water content compared with the control group (Figure 4A). The lipid content of adults at 20°C (*F* = 16.889, *df* = 3, *p* = 0.004) and 25°C (*F* = 16.889, *df* = 3, *p* = 0.000) was significantly higher than those of the control group (28°C). There was no significant difference in lipid content between 15°C and 28°C (Figure 4C).

After short-term exposure, the water and lipid content of adults at 0°C were significantly higher than those at 4°C (water content: *df* = 2, *p* = 0.011; lipid content: *F* = 4.542, *df* = 2, *p* = 0.028), but there was no significant difference between 0°C or 4°C and the control group (Figures 4B, D). The water content of adults at 4°C was significantly lower than that of the control group (28°C) (*df* = 2, *p* = 0.020) (Figure 4B). There was no significant difference in lipid content between 0°C and 4°C (Figure 4D).

**FIGURE 5:**
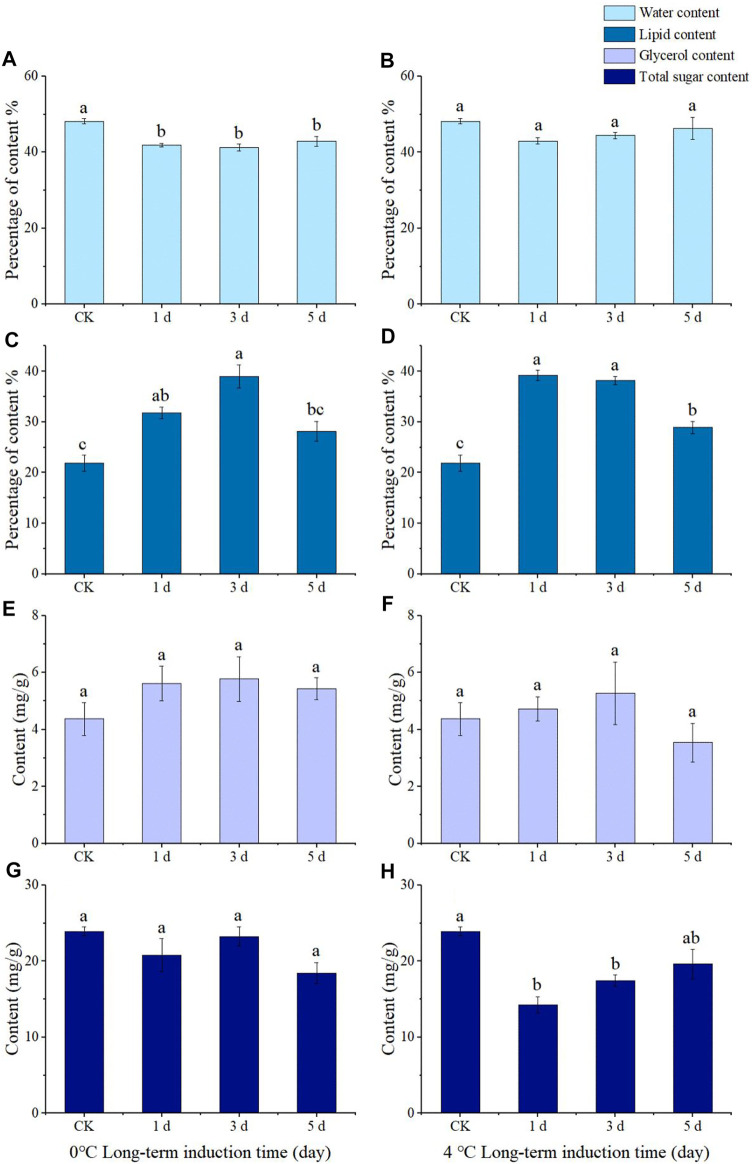
Water content **(A,B)**, lipid content **(C,D)**, glycerol content **(E,F)**, and total sugar content **(G,H)** of *Megabruchidius dorsalis* adults induced by long-term low temperature (0°C: A, C, E,G; 4°C: **(B,D,F,H)** for 1, 3, and 5 d.The data were Mean ± SE, and the different lowercase letters indicated a significant difference among time treatments under the same physiological indexes (Kruskal–Wallis test, One-Way ANOVA, Tukey, *p* < 0.05).

The body water content of adults induced at 0°C for 1, 3, and 5 d was significantly lower than that of the control group (28°C) (*F* = 13.173, *df* = 3, *p* = 0.000), yet there was no significant difference in body water content between 4 °C and conrol (Figures 5A, B). The lipid content of the adults treated at 0°C for 1 and 3 d was significantly higher than that of the control group (1 d: *F* = 15.396, *df* = 3, *p* = 0.007; 3 d: *F* = 15.396, *df* = 3, *p* = 0.000), and that of the adults treated at 0°C for 3 d was significantly higher than that of the adults treated for 5 d (*F* = 15.396, *df* = 3, *p* = 0.003) (Figure 5C). The lipid content in the control group was significantly lower than that in the treatment group at 4°C for 1, 3, and 5 d (*F* = 46.432, *df* = 3, *p* = 0.000), and the lipid content of adults induced at low temperature for 5 d was significantly lower than those induced for 1 d (*F* = 46.432, *df* = 3, *p* = 0.000) and 3 d (*F* = 46.432, *df* = 3, *p* = 0.000) (Figure 5D).

### 3.4 Glycerol and total sugar contents

After long-term low-temperature acclimation, content of 20°C and 25°C was significantly higher than that of the control group (*F* = 26.287, *df* = 3, *p* = 0.000), but the glycerol content of the adults at 15°C was not significantly different from that of the control group (Figure 4E). At 20°C (*F* = 4.027, *df* = 3, *p* = 0.017), the total sugar content was significantly higher than that of the control group (Figure 4G). At 15°C and at 25°C, there was no significant difference in the values measured in the CK group (Figure 4G).

The contents of glycerol and total sugar at 0°C and 4°C were not significantly different from those of the control group, and there was no significant difference in the contents of glycerol and total sugar between 0°C and 4°C (Figures 4F, H).

The results of the long-term induction showed no significant difference in glycerol content compared to CK after being treated at 0°C or 4°C for 1, 3, or 5 d (Figures 5E, F). The total sugar content in the control group at 4°C was significantly higher than that in the treatment group at 1 d (*F* = 11.454, *df* = 3, *p* = 0.002) and 3 d (*F* = 11.454, *df* = 3, *p* = 0.021) (Figure 5H), yet there was no significant difference in the total sugar content of the adults treated at 0°C for 1, 3, and 5 d (Figure 5G).

### 3.5 Correlation among the indexes after long-term low temperature acclimation of *M. dorsalis* adults

After long-term low temperature acclimation, there was a significant positive correlation between temperature and SCP, but a significant negative correlation between temperature with water content and total sugar content (Table 2). The SCP exhibited a significant negative correlation with glycerol content and total sugar content, while the glycerol content and total sugar content were both positively correlated with lipid content (Table 2).

**TABLE 2:** Spearman correlation coefficient matrix among the indexes after long-term low-temperature acclimation of *Megabruchidius dorsalis* adults.

	Temperature	SCP	Water content	Lipid content	Glycerol content	Total sugar content
Temperature	1					
SCP	0.66**	1				
Water content	−0.733***	−0.388	1			
Lipid content	0.109	−0.385	−0.443	1		
Glycerol content	−0.271	−0.608**	−0.127	0.722***	1	
Total sugar content	−0.473*	−0.552*	0.25	0.564**	0.671**	1

The asterisk (*) indicated a significant correlation among the indexes, * means *p* < 0.05, * * means *p* < 0.01, * * * means *p* < 0.001.

## 4 Discussion

### 4.1 Supercooling point and low lethal temperature

Supercooling is a significant property related to insect cold tolerance. The lower the SCP or FP, the higher the cold tolerance, and the cold tolerance also partly influences insect distribution (Pei et al., 2020). In general, freeze avoidance is the primary cold tolerance strategy used by insect species in temperate regions of the northern hemisphere (Sinclair and Chown, 2005). The cold tolerance of insects is impacted by both low temperatures and low temperature acclimation time (Salt, 1961). In the present study, there was no significant difference in SCPs between female and male adults of *M. dorsalis* after the three treatments, but the SCP of male *Callosobruchus chinensis* was significantly lower than that of female adults (Song, 2017). Insects can enhance their resistance to cold by either short-term (Chen et al., 1987) or long-term cold exposure (Danks, 1996). Cold acclimation allows insects to withstand cold conditions by lowering their SCP (Fuller, 2004; Fujikawa et al., 2018). The SCP and FP of *M. dorsalis* adults acclimated to low temperatures for a long time decreased with decreasing temperature (28°C–15°C), and SCP exhibited a significant positive correlation with temperature. Before overwintering, insects undergo a process of gradual cooling, during which they are physiologically prepared to cope with the cold winter temperatures (Fujikawa et al., 2018). The SCP and FP of *M. dorsalis* adults treated with short-term low-temperature exposure and long-term low-temperature induction at 0°C and 4°C were significantly lower than those at 28°C (CK). Both long-term and short-term low-temperature exposure could enhance the cold tolerance of *M. dorsalis* adults, which has similar results in *Sirex noctilio* (Li et al., 2021), *Ips typographus* (Koštál et al., 2011), and *Meligethes aeneus* (Hiiesaar et al., 2011). This suggests that *M. dorsalis* adults, similar to the insects mentioned above, may increase biofluid concentrations to decrease the SCP.

The LT_50_ of *M. dorsalis* in the present study (-11.36°C) was in the range of maximum (-5.8°C) and minimum (-15°C) SCP, and the LLT (-19.48°C) was much lower than the SCP (-10.62°C). The cold tolerance strategy of *M. dorsalis* could be characterized as freeze avoidance from separate measurements of SCP and LLT. Freeze-avoidance species may be susceptible to conditions that promote ice formation (Sinclair et al., 2015). The monthly mean minimum temperature in Zhengzhou in December, January, and February were lower than those in Guiyang, which might be the reason why the damage caused by *M. dorsalis* was more severe in Guizhou than that in Henan province.

### 4.2 Relationship between cryoprotectants and cold tolerance in *M. dorsalis* adults

Most overwintering insects reduce the damage of biofluids freezing by cryoprotective dehydration, increasing biofluid concentrations, decreasing the SCP, or reducing metabolic activity and energy consumption to increase survival at low temperatures (Koštál et al., 2011; Zhao et al., 2019). The water content of *M. dorsalis* adults at 15°C was significantly higher than that of the control group (at 28°C) under long-term temperature acclimation, which may be caused by differences in *M. dorsalis* behavior (crawling around at 28°C, while crouching at 15°C, personal observation) in this study. The water content was significantly lower than in the control group after short-term cold exposure at 4°C, suggesting that *M. dorsalis* adults might alleviate the damage caused by cold by reducing the water content in their bodies. However, after long-term cold acclimation, the influence of water content on *M. dorsalis* adults’ cold tolerance was not significant, similar to results from *Streltzoviella insularis* (Pei et al., 2020).

During overwintering, insects can regulate chemicals related to cold resistance (water, fat, carbohydrates, etc.) in the body through physiological and biochemical reactions and convert chemicals such as sugars into fat (Koštál et al., 2011). This phenomenon may occur before the overwintering onset and during the preparation phase. Fatty compounds can also be hydrolyzed into antifreeze agents such as glycerol to improve cold tolerance, such as in *Dendroctonus armandi* (Wang et al., 2017) and *A. glabripennis* (Feng et al., 2014). After long-term acclimation at 20°C and 25°C, the lipid content of *M. dorsalis* adults was significantly higher than that of the control group, which indicated that *M. dorsalis* adults could tolerate cold conditions by accumulating lipids, similar to the effects of lipid accumulation seen in *A. glabripennis* (Feng et al., 2016). Under the same low-temperature conditions, the lipid content of *M. dorsalis* adults treated for 3 d was significantly higher than those treated for 5 d, indicating that the duration of low-temperature exposure also affected the lipid content.

Glycerol and sugar are important cryoprotectants in insects (Fuller, 2004; Denlinger and Lee, 2010). After acclimation at 20°C, the contents of glycerol and total sugar in *M. dorsalis* adults were significantly higher than those in the control group, and the glycerol content was positively correlated with the lipid and total sugar content, suggesting that the adults resisted cold by accumulating energy and adding antifreeze protectants, which is consistent with the overwintering characteristics of *Pityogenes chalcographus* (Koštál et al., 2014). However, there was no significant difference between the short-term low temperature exposure group and the control group, possibly due to the lack of enough time for glycerol accumulation. After long-term induction at 4°C, the total sugar content of *M. dorsalis* adults treated for 1 d and 3 d was significantly lower than that of the control group. This might reflect the transformation of total sugar in *M. dorsalis* adults into low molecular-weight cryoprotectants, as seen in studies on cold hardiness in *S. insularis* (Qin et al., 2019). In addition, insects regulate cold tolerance by accumulating antifreeze proteins, heat shock proteins, and other proteins related to cold tolerance (Chang et al., 2019).

The cold-resistant strategy of *M. dorsalis* adults is freeze avoidance. The SCP and LLT of *M. dorsalis* adults at 28 °C were -10.62°C and -19.48°C, respectively. The water content of *M. dorsalis* adults after exposure at 4 °C for 2 hours was significantly lower than that of the control group. The contents of glycerol and total sugars in the long-term temperature acclimation group (20 °C) were significantly higher than those in the control group (28 °C). The SCP of *M. dorsalis* adults changed along with environmental temperatures, and the SCP of the long-term acclimation group was positively correlated with environmental temperature, but the SCP was negatively correlated with glycerol content. The glycerol content was significantly positively correlated with the lipid and total sugar content. *M. dorsalis* adults may maintain biofluids in a supercooled state by accumulating cryoprotectants (increasing biofluid concentrations) and cryoprotective dehydration to reduce freezing damage. In this study, the effects of environmental temperature and some metabolic components on the cold resistance of adults of *M. dorsalis* were investigated, but the physiological and molecular mechanisms of the cold hardiness of *M. dorsalis* have not been studied. Future research should focus on the cold tolerance mechanisms of *M. dorsalis*.
